# Pathophysiology of type 2 diabetes in sub-Saharan Africans

**DOI:** 10.1007/s00125-022-05795-2

**Published:** 2022-09-27

**Authors:** Julia H. Goedecke, Amy E. Mendham

**Affiliations:** 1 Biomedical Research and Innovation Platform and Non-Communicable Diseases Research Unit, South African Medical Research Council, Cape Town, South Africa; 2 South African Medical Research Council/WITS Developmental Pathways for Health Research Unit (DPHRU), Department of Paediatrics, School of Clinical Medicine, Faculty of Health Sciences, University of the Witwatersrand, Johannesburg, South Africa; 3 Health through Physical Activity, Lifestyle and Sport Research Centre (HPALS), FIMS International Collaborating Centre of Sports Medicine, Division of Physiological Sciences, Department of Human Biology, Faculty of Health Sciences, University of Cape Town, Cape Town, South Africa

**Keywords:** Beta cell function, Epigenetics, Ethnicity, Genetics, Hyperinsulinaemia, Infectious diseases, Insulin resistance, Insulin sensitivity, Obesity, Review, Social determinants

## Abstract

Sub-Saharan Africa (SSA) is the region with the highest projected rates of increase in type 2 diabetes (129% by 2045), which will exacerbate the already high prevalence of type 2 diabetes complications and comorbidities in SSA. In addition, SSA is grappling with poverty-related health problems and infectious diseases and is also undergoing the most rapid rates of urbanisation globally. These socioenvironmental and lifestyle factors may interact with genetic factors to alter the pathophysiological sequence leading to type 2 diabetes in sub-Saharan African populations. Indeed, current evidence from SSA and the diaspora suggests that the pathophysiology of type 2 diabetes in Black Africans is different from that in their European counterparts. Studies from the diaspora suggest that insulin clearance is the primary defect underlying the development of type 2 diabetes. We propose that, among Black Africans from SSA, hyperinsulinaemia due to a combination of both increased insulin secretion and reduced hepatic insulin clearance is the primary defect, which promotes obesity and insulin resistance, exacerbating the hyperinsulinaemia and eventually leading to beta cell failure and type 2 diabetes. Nonetheless, the current understanding of the pathogenesis of type 2 diabetes and the clinical guidelines for preventing and managing the disease are largely based on studies including participants of predominately White European ancestry. In this review, we summarise the existing knowledge base and data from the only non-pharmacological intervention that explores the pathophysiology of type 2 diabetes in SSA. We also highlight factors that may influence the pathogenesis of type 2 diabetes in SSA, such as social determinants, infectious diseases and genetic and epigenetic influences.

**Graphical abstract:**

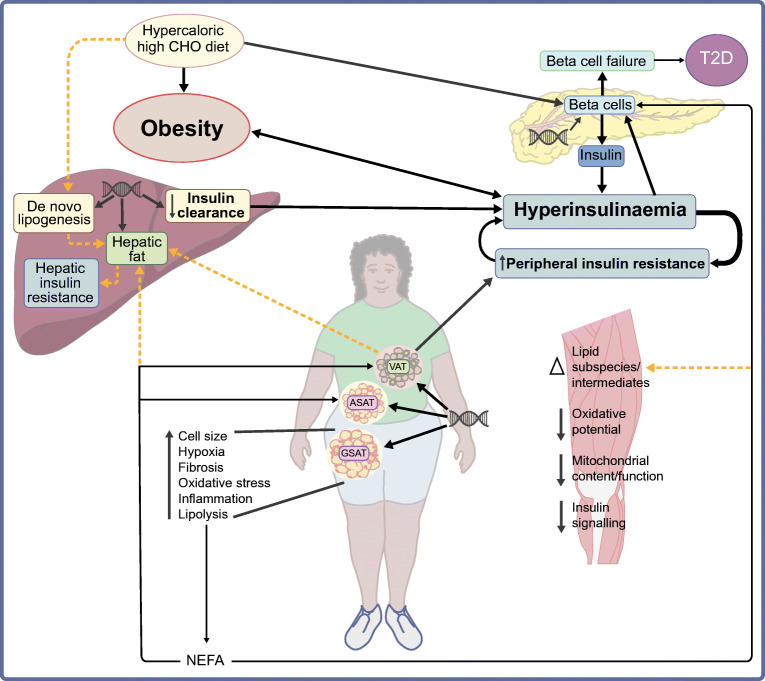

**Supplementary Information:**

The online version contains a slideset of the figures for download available at 10.1007/s00125-022-05795-2.

## Introduction

Global estimates for the prevalence of type 2 diabetes are as high as 9.3%, affecting 463 million people [1]. While the prevalence of type 2 diabetes is lowest in the sub-Saharan Africa (SSA) region (4.7%), this varies by country, with the highest number of people with type 2 diabetes residing in more affluent countries [1]. SSA is also the region with the highest burden of infectious diseases, which also impacts on type 2 diabetes risk [2]. Notably, SSA is projected to have the greatest rates of increase in type 2 diabetes (129% by 2045) compared with other IDF regions [1]. This will exacerbate the already high prevalence of type 2 diabetes complications and comorbidities in SSA [3], placing additional strain on the already overburdened healthcare systems.

Despite the increasing projected rates of type 2 diabetes and other non-communicable diseases, SSA is still grappling with poverty-related health problems and is also undergoing the most rapid rates of urbanisation globally. These socioenvironmental and lifestyle factors may interact with genetic factors to alter the pathophysiological sequence leading to type 2 diabetes in sub-Saharan African populations. Indeed, current evidence suggests that the pathophysiology of type 2 diabetes in Black Africans is different from that in their European counterparts [4]. Nonetheless, the current understanding of the pathogenesis of type 2 diabetes and the clinical guidelines for preventing and managing the disease are largely based on studies including participants of predominately White European ancestry. This review aims to consolidate the current knowledge base on the mechanisms underlying type 2 diabetes risk in Black African populations living in SSA. As the pathophysiology of type 2 diabetes in diasporic Africans has been extensively reviewed [5–9], and due to the high degree of genetic admixture and different environmental exposures [10], we will focus primarily on studies including sub-Saharan African populations, which are under-represented in the literature. The review will highlight commonalities and differences between sub-Saharan African and diasporic populations, and importantly identify unique characteristics that influence the pathogenesis of type 2 diabetes in SSA, such as social determinants, infectious diseases and genetic/epigenetic factors.

## Known mechanisms relating to the pathophysiology of type 2 diabetes in sub-Saharan Africans

**Fig. 1:**
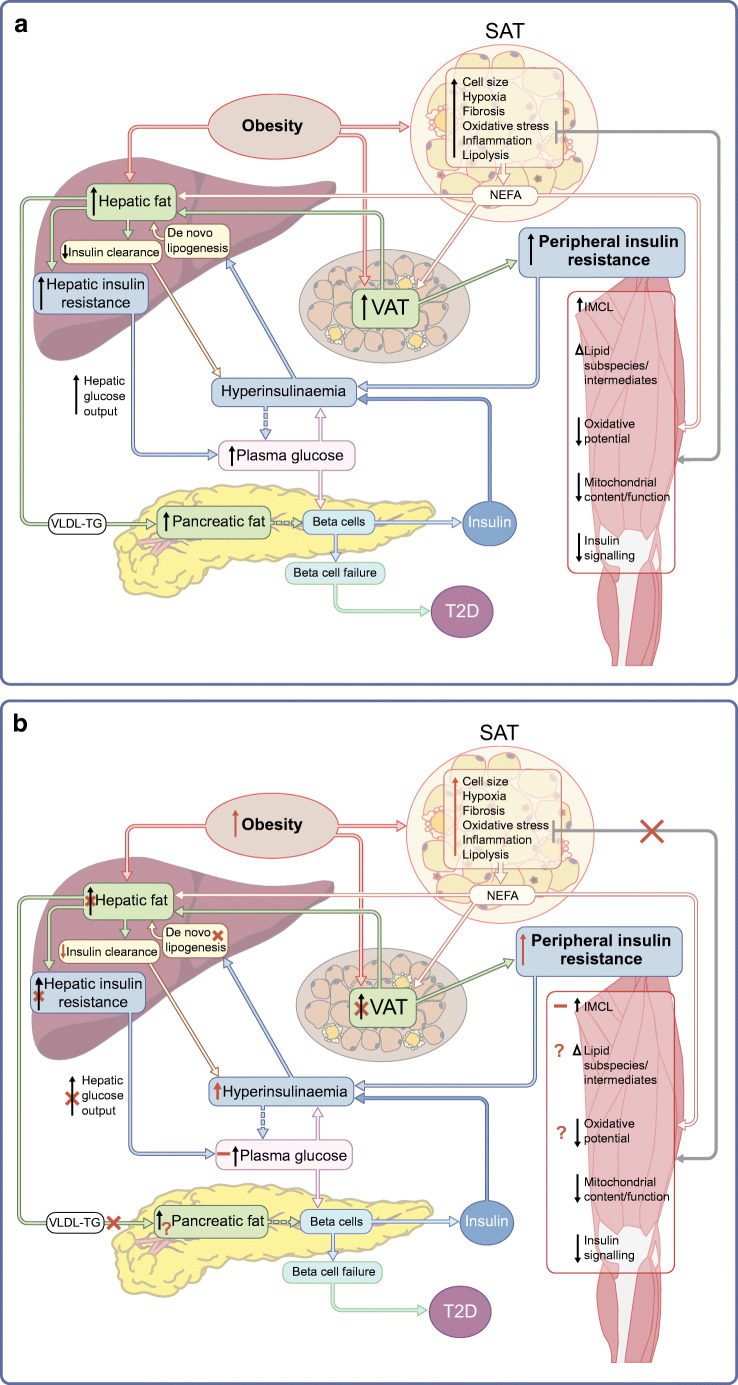
(**a**) Conventional paradigm of type 2 diabetes showing that insulin resistance is the primary abnormality, which is accompanied by a compensatory increase in insulin secretion, coupled with a decrease in insulin clearance to maintain normoglycaemia, until beta cell exhaustion ensues and hyperglycaemia develops. This conventional paradigm posits that increasing obesity leads to adipocyte hypertrophy, oxidative stress, fibrosis and macrophage recruitment and the release of adipokines and inflammatory mediators. This increased inflammatory state, together with reduced adipocyte adipogenic capacity, leads to adipocyte lipolysis and the overflow of excess NEFAs to visceral adipose tissue (VAT) and other ectopic sites (e.g. liver, muscle and pancreas). The increased inflammation and ectopic fat accumulation results in reduced hepatic and peripheral insulin sensitivity [12]. This model is based on studies including predominately populations of European descent. (**b**) Comparison of the conventional model of the pathogenesis of type 2 diabetes with findings from studies of Black Africans from SSA. These findings relate to studies in predominately Black African women and show that, compared with White Europeans, Black Africans have a higher prevalence of obesity but present with a phenotype of low levels of VAT and high levels of abdominal and gluteo-femoral subcutaneous adipose tissue (SAT). Notably, Black Africans have lower insulin sensitivity and present with hyperinsulinaemia, characterised by high insulin secretion and reduced hepatic insulin clearance. While little is known about pancreatic fat content, levels of intramyocellular lipids (IMCL) do not differ by ethnicity, but hepatic fat content is lower in Black Africans, which corresponds to lower de novo lipogenesis and lower circulating VLDL-TG concentrations. Lower hepatic fat accumulation is associated with higher hepatic insulin sensitivity and lower hepatic glucose output, and accordingly the prevalence of IFG is relatively low compared with that in White Europeans. The characteristics of gluteal SAT shown in the figure are amplified by obesity. While higher inflammation levels are observed in SAT of Black Africans, this is not associated with insulin sensitivity as in White Europeans. Dotted lines indicate an inverse relationship; red crosses identify characteristics that are lower in Black Africans; red arrows emphasise a stronger relationship in Black Africans; – indicates no differences compared with White Europeans; ? indicates uncertainty. T2D, type 2 diabetes; VLDL-TG, VLDL-triacylglycerol. This figure is available as part of a downloadable slideset

While it is well accepted that insulin resistance and beta cell dysfunction contribute to the pathogenesis of type 2 diabetes (Fig. 1a), there is still debate regarding the pathogenic sequence of events leading to type 2 diabetes [11]. The conventional paradigm is that insulin resistance is the primary defect, resulting in compensatory hyperinsulinaemia and leading eventually to beta cell exhaustion and type 2 diabetes [12]. However, there is increasing support for the theory that hyperinsulinaemia may be the distinct first event in the pathogenesis of type 2 diabetes [11], with the primary hyperinsulinaemic factors being posited as hypersecretion of insulin from the beta cells [11, 13] and/or reduced hepatic insulin clearance [14].

### Hyperinsulinaemia as the primary event in the pathophysiology of type 2 diabetes

**Fig. 2:**
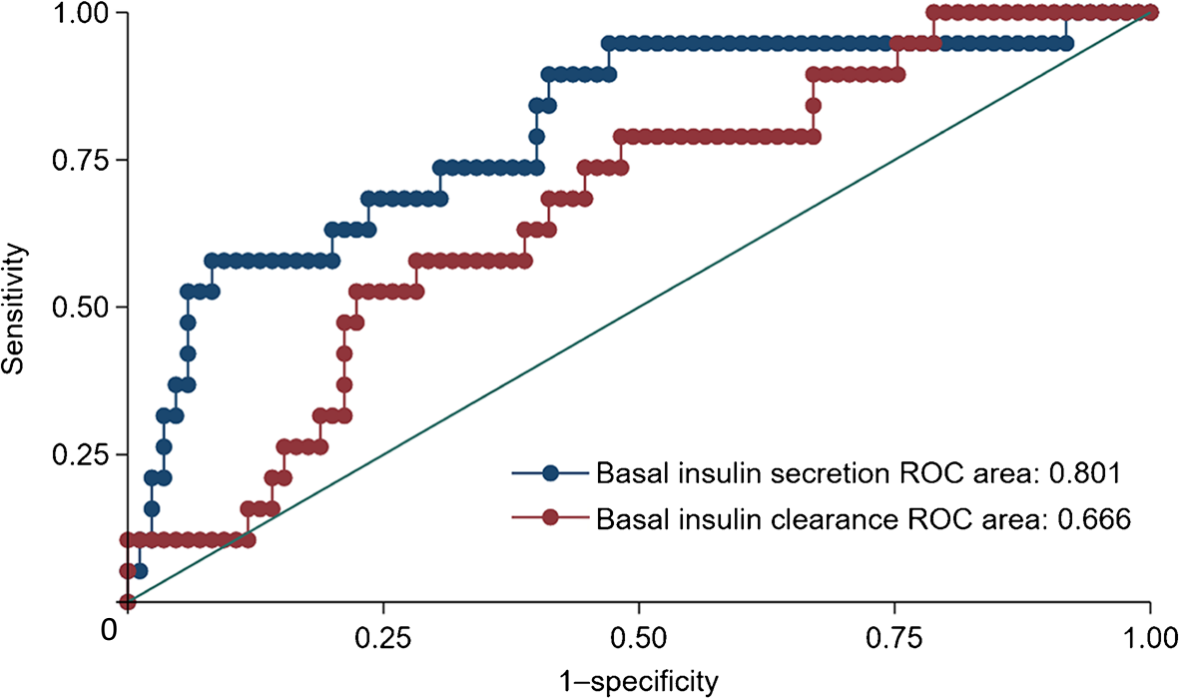
Comparison of receiver operating characteristic (ROC) curves of basal insulin secretion and clearance to predict dysglycaemia in middle-aged Black South African women 1.5 years later (Mtintsilana and Goedecke et al, unpublished). This figure is available as part of a downloadable slideset

Studies from SSA and the diaspora have consistently shown that the most characteristic feature in the pathophysiology of type 2 diabetes in Black African populations is the presentation of hyperinsulinaemia (Fig. 1b), as reviewed previously [7, 9]. Compared with White Europeans, Black Africans with normal glucose tolerance (NGT), impaired fasting glucose (IFG) or impaired glucose tolerance (IGT) present with hyperinsulinaemia [9], characterised by higher insulin secretion and lower insulin clearance, which is largely independent of differences in adiposity and insulin sensitivity [9, 15, 16]. This phenotype is observed in indigenous and diasporic Black African adults and children [17], suggesting that this trait is highly conserved [16]. However, it is still not clear if this hyperinsulinaemia is due to exaggerated beta cell function or low hepatic insulin clearance. While most studies to date have relied on cross-sectional designs, the prevailing evidence from West Africa, the UK and the USA has shown that low hepatic insulin clearance is the main driver of hyperinsulinaemia in adults [16, 18, 19], with insulin clearance being proposed as the primary defect underlying the development of type 2 diabetes [8, 14]. This is in direct contrast to our recent evidence indicating that elevated insulin secretion was more closely associated with hyperinsulinaemia than low hepatic insulin clearance in Black South African women with obesity [20]. In preliminary analyses of a small (*n*=112) prospective cohort of middle-aged Black South African women with NGT, we showed that insulin secretion, and not insulin clearance, independently predicted incident dysglycaemia 1.5 years later (Mtintsilana and Goedecke et al, unpublished; Fig. 2). The few other studies from SSA have relied on a cross-sectional design, with some contradictory findings in relation to these putative pathophysiological links. Amoah et al [21] showed that, despite similarities in insulin sensitivity, healthy NGT first-degree relatives of Ghanaians with type 2 diabetes had higher insulin and C-peptide responses to both oral and intravenous glucose than healthy NGT control groups without a family history of type 2 diabetes. These findings suggest that hyperinsulinaemia may be a primary factor in the aetiology of type 2 diabetes in Black Africans. The presentation of type 2 diabetes in this population was characterised by severe beta cell dysfunction, and to a lesser extent a decrease in insulin sensitivity [21], which is consistent with the findings from the Africans in America study, which includes Black Africans born in SSA and currently living in the USA [22]. The study showed that beta cell failure rather than insulin resistance was the main aetiological factor in 62% of Africans with IGT [23]. This corresponds to the findings from a cross-sectional study among Tanzanian adults, which showed that beta cell dysfunction and insulin resistance were associated with a higher risk of IGT and type 2 diabetes, and that beta cell dysfunction was the most important contributor to type 2 diabetes [23]. In contrast, results of the Research on Obesity and Diabetes among African Migrants (RODAM) study showed that insulin resistance (HOMA-IR) and not beta cell dysfunction accounted for geographical differences in IFG between rural and urban/migrant Ghanaians without type 2 diabetes [24]. However, this was a cross-sectional study with fasting glucose as the only marker of dysglycaemia, and it is well known that dysglycaemia in Black African populations overwhelmingly presents as elevated 2 h glucose and not as elevated fasting glucose [25]. Glucose tolerance is not only dependent on insulin-mediated glucose uptake, but also depends on the ability of glucose to mediate its own uptake (glucose effectiveness), which accounts for ~45–65% of glucose disposal [26]. However, glucose effectiveness does not differ by ethnicity [15] and is not a characteristic of type 2 diabetes in Black Africans [21]. We propose that, in SSA, hyperinsulinaemia due to a combination of both increased insulin secretion and reduced hepatic insulin clearance may be the primary aetiological factor, which promotes obesity [17] and insulin resistance, exacerbating the hyperinsulinaemia and eventually leading to beta cell failure and type 2 diabetes.

### The role of adipose tissue in the pathogenesis of insulin resistance and type 2 diabetes

Regardless of the sequence of events, studies in SSA and the diaspora have consistently shown that Black Africans have lower insulin sensitivity than their White European counterparts [5, 6, 8, 15, 27]. To date, most of the work in understanding the high prevalence of insulin resistance in Black Africans from SSA has focused on adipose tissue. This was largely driven by the paradoxical but consistent finding that, despite lower whole-body insulin sensitivity, Black Africans have less visceral adipose tissue (VAT) and more abdominal and gluteo-femoral subcutaneous adipose tissue (SAT) than their BMI-matched White European counterparts [28, 29], which is consistent with the pattern seen in diasporic Africans [5, 17].

Accordingly, the veracity of the traditional paradigm of the pathogenesis of type 2 diabetes, with obesity and adipose tissue expansion being the initial manifestation of insulin resistance [12] (Fig. 1a), was tested (Fig. 1b). We found that, compared with normal-weight women, the expression of adipogenic and lipogenic genes was reduced in the gluteal depot to a greater extent in Black South African women with obesity than in White South African women with obesity [30], which corresponded to Black South African women having a greater proportion of large adipocytes in this depot. Further, the gluteal SAT of Black South African women with obesity exhibited higher expression of genes relating to hypoxia, fibrosis and inflammation than that of their White counterparts [31, 32]. These findings suggest that, despite a more ‘favourable’ body fat distribution, a lower capacity to store fat in the gluteo-femoral depot with increasing obesity is associated with reduced insulin sensitivity in Black South African women [30, 31]. Indeed, gluteal SAT of Black South African women with obesity exhibited increased mitochondrial respiration capacity and hydrogen peroxide production than abdominal SAT, suggestive of cellular stress related to an over flux of NEFA into the mitochondria [33]. The higher oxidative stress was associated with lower insulin sensitivity [33]. Surprisingly, the higher SAT inflammatory profile of Black South African women was not associated with their lower insulin sensitivity, as in White South African women [32]. These findings, together with another study including Black South African women with obesity showing regional differences in the transcriptome signatures between abdominal and gluteal SAT [34], suggest that there are differences in developmental processes regulating the expandability of distinct adipose tissue depots.

### The role of ectopic fat in the pathogenesis of type 2 diabetes

Despite evidence of low gluteal SAT expandability in Black South African women with obesity, there is consistent evidence from Africa and the diaspora showing lower ectopic fat deposition than in White Europeans [4, 8, 35]. While the evidence relating to intramyocellular and pancreatic fat is limited [20], there is robust evidence showing that Black Africans have lower hepatic fat accumulation than White Europeans [35, 36], which parallels their lower levels of VAT [28, 29]. Lower hepatic fat content in Black South African women with obesity corresponded to higher hepatic insulin sensitivity compared with their White counterparts [36], as well as lower estimated rates of de novo lipogenesis [37], consistent with findings in African Americans [38]. Accordingly, circulating triacylglycerol levels are lower in Black Africans and are not associated with reduced insulin sensitivity [39]. This suggests that, unlike in White European populations, de novo lipogenesis and hepatic fat accumulation are not early features of insulin resistance and type 2 diabetes in Black African populations. This is supported by our recent cross-sectional study in Black South African women with obesity in which we showed that higher VAT levels, and not pancreatic or hepatic fat, were associated with lower first-phase insulin secretion and higher hepatic insulin clearance [20]. Indeed, with increasing age and adiposity, Black African women have a greater relative propensity to accumulate VAT compared with abdominal or gluteo-femoral SAT [40]. Notably, both baseline and the change in VAT predicted incident type 2 diabetes in middle-aged Black South African women 13 years later [40]. This raises the question as to whether Black Africans are more sensitive to the effects of ectopic fat accumulation than their White European counterparts [36]. A recent study in Ghanaians, using fatty liver index as a proxy for liver fat, showed that the fatty liver index increases with increasing urbanisation and is associated with prevalent type 2 diabetes [41], which supports the latter hypothesis.

### Sex differences in the pathophysiology of type 2 diabetes

There is a sexual dimorphism in type 2 diabetes risk in Africans, which is clearly illustrated by a similar type 2 diabetes prevalence in sub-Saharan African men and women [1] despite vast differences in obesity prevalence (e.g. 41% vs 11% in South African women and men, respectively) [42]. The few studies including sex comparisons indicate that Black African women exhibit greater hyperinsulinaemia than Black African men [22, 43]. However, we argue that Black South African men are at a higher risk for type 2 diabetes than Black South African women for the following reasons: (1) when adjusting for differences in body fat, men have lower insulin sensitivity, insulin secretion and beta cell function, while insulin clearance did not differ by sex; (2) men have a less ‘favourable’ body fat distribution, with more VAT and less abdominal and gluteo-femoral SAT; (3) there is a stronger relationship between total and central adiposity and type 2 diabetes risk in men; (4) men have a lower ‘protective’ effect of leg fat mass on beta cell function than women [43]. These disparities may be driven by the obvious effects of sex hormones, but there are no studies to our knowledge that have explored these effects in SSA. In terms of sex differences in lifestyle factors, studies in SSA have shown that men are more likely to smoke, consume alcohol and participate in more moderate-to-vigorous physical activity (MVPA) than women [44, 45], which may confound these associations. Indeed, we recently showed that MVPA was associated with lower type 2 diabetes risk in men, whereas light physical activity was associated with reduced type 2 diabetes risk in women [46]. When interrogating the relevance of dietary intake, we showed that, although nutrient patterns did not differ between men and women, the strength of the association between the animal-driven nutrient pattern and BMI was greater in men than in women. In contrast, the plant-driven pattern, characterised by the intake of refined carbohydrates, was associated with increases in abdominal SAT in women but not in men [47]. We postulated that hyperinsulinaemia observed in Black African women compared with men may drive this relationship. However, to our knowledge, there are no longitudinal studies exploring the pathogenesis of type 2 diabetes in sub-Saharan African men. Further, most studies exploring the pathophysiology of type 2 diabetes in sub-Saharan African populations are focused on women or include more women than men, which may bias our interpretation of the results. Longitudinal and intervention studies are thus key to illuminating the full aetiology and pathogenic sequence of type 2 diabetes in both Black African men and women.

## Lifestyle interventions to inform our understanding of the pathophysiology of type 2 diabetes

**Fig. 3:**
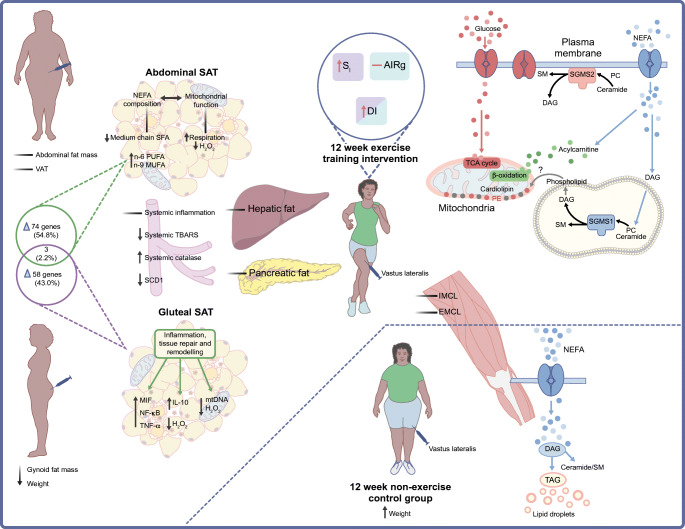
Schematic diagram indicating the changes in response to a 12 week combined aerobic and resistance exercise training intervention in Black South African women with obesity. The exercise intervention resulted in an increase in insulin sensitivity (S_I_) but no change in acute insulin response to glucose (AIRg), with a corresponding increase in the disposition index (DI), an estimate of beta cell function [49]. Ectopic lipid content, measured in the liver, pancreas and skeletal muscle (intramyocellular lipids [IMCL] and extramyocellular lipids [EMCL]), did not change in response to the intervention, but functional changes in skeletal muscle and adipose tissue were evident. Exercise training resulted in content-driven improvements in mitochondrial function that were associated with changes in lipid intermediates [57]. With an increase in body weight, skeletal muscle triacylglycerol subspecies and lipid intermediates (ceramides and sphingomyelins) were increased in the control group. However, the changes in skeletal muscle lipid metabolism in both the exercise group and the control group did not correspond to changes in IMCL or EMCL. Exercise training resulted in a small but significant decrease in body weight and gynoid fat mass (% of total fat mass), with a greater reliance on fat oxidation at baseline promoting the reduction in gynoid fat mass [98]. Using a transcriptome approach, we showed that exercise training resulted in a change in the expression of 58 genes in the gluteal SAT, and these differed from the 74 genes whose expression was changed in abdominal SAT [34]. Within the gluteal SAT, these genes were mainly related to immune and inflammatory responses and lipid metabolism, whereas in the abdominal SAT these genes were related to muscle-associated processes [34]. Commensurate with these findings, we reported a higher inflammatory gene expression profile (TNF-α, IL-10, MIF and NF-κB mRNA) in the gluteal (and not abdominal) SAT following exercise training, which may reflect tissue remodelling related to the decrease in gynoid fat mass [56]. Gluteal SAT was the depot that showed the most consistent reductions in H_2_O_2_ emissions, as a marker of reactive oxygen species (ROS) production [33]. These results further support the systemic adaptations, which show a decrease in circulating thiobarbituric acid reactive substances (TBARS), a by-product of lipid peroxidation by ROS, with a simultaneous increase in circulating catalase, a reflection of antioxidant enzyme activity [56]. Although exercise training did not change abdominal fat content, abdominal SAT mitochondrial respiration and coupling increased and alterations in the fatty acid profile were observed [33, 99]. These findings show changes in the functional capacity of abdominal SAT and highlight major depot-specific differences that reflect the heterogeneous capacity of SAT to adapt to behavioural changes such as exercise training, which indirectly influence signalling pathways that regulate fat distribution and insulin dynamics. Finally, we showed a decrease in estimated stearoyl-CoA desaturase (SCD1) activity, a marker of de novo lipogenesis, which was associated with lower liver fat levels [99]. Arrows indicate changes; – indicates no change in response to the intervention. DAG, diacylglycerol; MIF, macrophage migration inhibitory factor; mtDNA, mitochondrial DNA; MUFA, monounsaturated fatty acid; PC, phosphatidylcholine; PUFA, polyunsaturated fatty acid; SFA, saturated fatty acid; SGMS1, sphingomyelin synthase 1; SGMS2, sphingomyelin synthase 2; SM, sphingomyelin; TAG, triacylglycerol; TCA, tricarboxylic acid. This figure is available as part of a downloadable slideset

Lifestyle interventions are used as non-pharmacological models to understand the pathophysiology of type 2 diabetes. Specifically, these models have focused on improving insulin sensitivity and/or beta cell function via exercise training or dietary-induced weight loss [48, 49]. This research approach has primarily focused on populations of White European descent, with limited data on Africans and the diaspora [50, 51] and only one study in SSA [52]. The results of the lifestyle intervention studies in African Americans have been previously reviewed [17, 53] and it was concluded that African Americans were resistant to weight change compared with their White American counterparts. The study in SSA was an RCT designed to examine the mechanisms underlying the changes in insulin sensitivity and secretion in response to a 12 week exercise (combined aerobic and resistance) intervention in young Black South African women with obesity [52]. The study aimed to identify causal pathways underlying the high prevalence of insulin resistance and risk for type 2 diabetes, while targeting specific areas for therapeutic intervention (Fig. 3).

Studies that have shown an improvement in insulin sensitivity after exercise training have mainly reported a reduction in insulin response [54, 55], with exercise intensity and volume influencing the degree of change [55]. The 12 week exercise intervention in Black South African women showed that the improvement in insulin sensitivity was not matched by a change in insulin response, characterised by insulin secretion or clearance [49]. This suggests that hyperinsulinaemia may not be a compensatory response to insulin resistance in Black Africans and the maintenance of hyperinsulinaemia may have attenuated exercise-induced lipolysis and weight loss. Indeed, we observed only a ~1 kg weight loss in response to the 12 week moderate/high-intensity exercise training intervention [49]. We also did not observe any changes in liver, muscle or pancreatic fat, suggesting that the improvement in insulin sensitivity in response to exercise training may be independent of ectopic lipids [49]. Further, the improvement in insulin sensitivity was not related to the other traditional mechanisms underlying insulin resistance, including body fat distribution, adipose tissue and skeletal muscle function, and systemic inflammation [33, 49, 56, 57]. These findings provide further evidence that the pathogenesis of type 2 diabetes in Black Africans may be different from that in Europeans [4].

**Fig. 4:**
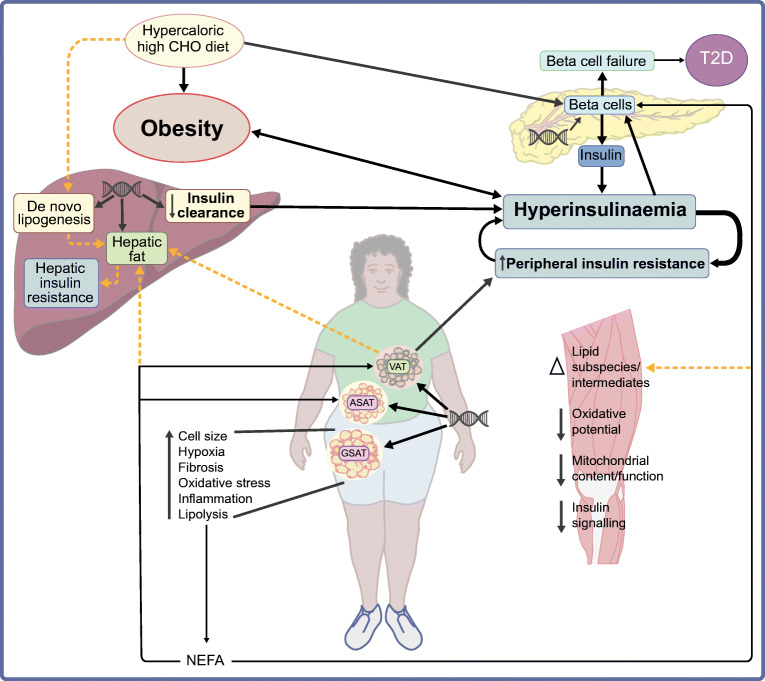
Proposed model of the pathogenesis of type 2 diabetes in sub-Saharan African populations. Pathogenesis is characterised by hyperinsulinaemia due to reduced insulin clearance and hypersecretion of insulin, which is probably driven by a genetic predisposition and lifestyle factors, in particular the consumption of a hypercaloric, high-carbohydrate (CHO) diet. Hyperinsulinaemia reduces skeletal muscle insulin sensitivity, creating a negative feedback loop, exacerbating hyperinsulinaemia and obesity and eventually leading to beta cell failure and the development of type 2 diabetes. Black African women present with a phenotype of low VAT and high gluteo-femoral SAT, which is probably genetically determined. The large gluteo-femoral SAT depot acts as a reservoir for excess fatty acids, but eventually the adipogenic capacity is exceeded, and changes within the gluteo-femoral SAT result in an increase in lipolysis and release of NEFAs, which may further stimulate beta cell function. The excess NEFAs are redistributed to the abdominal region and ectopic sites (liver and muscle). The increase in VAT and/or liver fat from a lower baseline and the greater sensitivity to the effects of these depots exacerbate the insulin-resistant state. Insulin resistance within skeletal muscle is not characterised by increased intramyocellular lipids, but rather by changes in lipid intermediates and subspecies. These changes are associated with decreased mitochondrial content and oxidative capacity and changes in insulin signalling pathways. The pathophysiology of type 2 diabetes in Black African men differs from that in women. Compared with women with the same level of body fatness, men have lower insulin sensitivity, insulin secretion and beta cell function, and show a stronger relationship of total and central adiposity with type 2 diabetes risk. Men with type 2 diabetes often present with a phenotype of low BMI (<25 kg/m^2^) and low insulin secretion and beta cell failure. Exposure to infectious disease may further influence the pathogenesis of type 2 diabetes and further impact the risk for type 2 diabetes. Solid lines represent the direction of relationships and thicker lines represent stronger relationships; dotted orange lines show attenuated relationships. ASAT, abdominal subcutaneous adipose tissue; GSAT, gluteo-femoral subcutaneous adipose tissue; T2D, type 2 diabetes. This figure is available as part of a downloadable slideset

We propose a model of the pathogenesis of type 2 diabetes in Black Africans from SSA (Fig. 4) that needs to be tested in longitudinal and intervention studies. We suggest that interventions designed specifically to reduce hyperinsulinaemia and induce greater reductions in adiposity may be more appropriate to investigate the pathogenesis of type 2 diabetes in Black Africans. Very low energy (very low calorie diets [VLCDs]) and low-carbohydrate diets are associated with reduced insulin secretion [48, 58] and may be more appropriate for populations with hyperinsulinaemia. Indeed, a study from the USA showed that African American women lost more weight in response to a lower-carbohydrate diet than a lower-fat diet, whereas there was no difference in weight loss between the two diets in European women [59]. The only study to use a VLCD to reverse type 2 diabetes in a population of African descent was performed in Barbados. This study reported less weight loss than similar studies in Europe [60]. Nonetheless, the study found that 10 kg weight loss was associated with remission in 60% of participants at 8 weeks and in 38% of participants at 8 months; however, it did not explore the putative underlying mechanisms. The attenuated weight loss in these diasporic populations may reflect the consequences of hyperinsulinaemia in African populations [17] and/or differences in sociocultural factors, which may influence adherence to interventions. To date, there are no studies in SSA that have assessed the effects of dietary interventions on the pathophysiology of type 2 diabetes, and future research is warranted.

## Factors that may influence the pathogenesis of type 2 diabetes in sub-Saharan Africans

### Social determinants of type 2 diabetes

An analysis of the pathogenesis underlying type 2 diabetes risk would be incomplete without addressing the effects of syndemics and the social determinants of health. To understand the phenotype in Black Africans, it is key to understand the interplay between biology, disease clusters and lifestyle factors, as well as how these factors impact known mechanisms relating to the development of type 2 diabetes. Within SSA, rapid demographic, sociocultural and economic transitions are driving increases in risk factors for type 2 diabetes [61]. Indeed, the characteristics of people with type 2 diabetes differ between those living in urban settings and those living in rural settings [62, 63], with those in rural settings characterised by having a lower socioeconomic status (SES), younger age of onset, higher prevalence of reported childhood undernutrition and lower prevalence of traditional risk factors such as obesity [63]. In direct contrast to findings from Europe, level of education, which is the most stable and sensitive marker of SES, is positively associated with type 2 diabetes in SSA [62]. This relationship is likely mediated by the effects of early life experiences and/or effects of other lifestyle factors that impact type 2 diabetes risk, such as dietary intake and physical activity. While countries within SSA are still in the early stages of the nutrition transition [64], low dietary diversity and the high reliance on processed carbohydrate-rich staple diets [65, 66] in the context of hyperinsulinaemia may drive the increases in obesity, particularly in women [47].

Although the prevalence of obesity is increasing in SSA, undernutrition remains a problem, especially in young children and in adults with severe infections. Notably, childhood SES and early life nutritional status are associated with increased risk for type 2 diabetes in adults [67–69], with this amplified by subsequent catch-up growth or adult obesity [68, 69]. Exposure to chronic undernutrition is associated with low insulin production in both children [70] and adult men [71]. This corresponds with the presentation of the predominant form of atypical diabetes in SSA, identified as malnutrition-related diabetes mellitus [72]. This phenotype is highly prevalent (~30% of patients) and presents in people living in low socioeconomic circumstances and who have a low BMI and relative beta cell impairment [72, 73]. This presence of different subtypes of diabetes in SSA needs to be explored further in large phenotyping and genotyping studies. These studies need to consider traits most prevalent in SSA, for example, sickle cell disease, which is an inherited disorder characterised by structural changes in haemoglobin. While people with sickle cell trait (representing ~25% of the sub-Saharan African population) do not have symptoms of sickle cell disease, they are at higher risk of beta cell dysfunction, with the risk for type 2 diabetes exacerbated by anti-retroviral therapy (ART) [74].

### Infectious diseases

Fifty per cent of global deaths and disability-adjusted life-years due to infectious diseases are in SSA [75], with SSA having the greatest burden of HIV/AIDS (67.5% of all 37.9 million people living with HIV [PLWH]). In SSA, young women and adolescent girls accounted for 63% of all new HIV infections in 2020, of whom Black South African women/girls are disproportionately affected [76]. Since the successful roll-out of ART in SSA, there has been an associated rise in life expectancy and non-communicable diseases such as obesity and type 2 diabetes in PLWH, ensuring the collision of these disease clusters [77]. Indeed, protease inhibitors impair beta cell function by increasing apoptosis and oxidative stress, thereby decreasing insulin secretion [78]. While data from Africa are limited and there is a need for prospective longitudinal studies, a recent meta-analysis of studies of African populations showed no association between the prevalence of type 2 diabetes and HIV infection or ART [79]. Regardless, the occurrence of type 2 diabetes seen with first-generation ART has been partly resolved with newer drugs, but there may be residual effects of long-term exposure to multiple first-generation ARTs [80]. The ADVANCE study in South Africa has shown a ‘return to health’ weight gain in ART-naive men and women initiating the new first-line therapy in Africa (dolutegravir) compared with those initiating efavirenz [81]. However, this has raised concerns about the detrimental effects of weight gain and risk for metabolic abnormalities in an obesogenic environment. Notably, PLWH in Africa often present with other viral co-infections such as tuberculosis and hepatitis C, resulting in chronic low-grade inflammation that can further exacerbate the risk of developing type 2 diabetes. In addition to factors such as chronic inflammation and immune activation, PLWH in Africa commonly experience sociodemographic disparity and chronic malnutrition compared with HIV-uninfected people [71]. These factors combined may explain the higher prevalence of type 2 diabetes in PLWH.

In addition to HIV, other viruses may trigger type 2 diabetes development. Results from a meta-analysis have shown that hepatitis C infection, which has one of the highest reported seroprevalence rates in West Africa (~2.8%) [82], is associated with a ~1.7-fold increased risk for type 2 diabetes compared with non-infected control groups [83]. Additionally, there is evidence to suggest that *Human herpesvirus 8* infection is strongly linked to type 2 diabetes and, in particular, ketosis-prone type 2 diabetes [84], which frequently occurs in individuals of African origin and is characterised by an acute and reversible deficiency in insulin secretion. In SSA, *Human herpesvirus 8* infection is not related to a decrease in insulin sensitivity in patients with diabetes [85], but rather is associated with low insulin secretion [86], which is supported by an early in vitro study showing that *Human herpesvirus 8* can directly infect human pancreatic beta cells [84]. In contrast to these viruses, *Schistosoma* and geohelminth infections lower the risk of type 2 diabetes [87], with a recent study from Tanzania showing that *Schistosoma* infection was associated with higher beta cell function [88]. However, this was offset by HIV co-infection, as *Schistosoma* and geohelminth infections were associated with reduced beta cell function among PLWH and ART-naive individuals [88].

### Genetic and epigenetic factors

Genome-wide association studies (GWAS) have identified over 400 risk loci for type 2 diabetes. However, most of these studies have been conducted in European populations, and studies conducted in African populations have predominately included African American populations [89]. Although these studies are informative, African Americans are admixed (~20% European ancestry) and their environmental exposures also differ from those in people living in SSA. Africans harbour a far greater amount of genetic diversity and recently about 3 million variants from this population group were reported to be missing from publicly available databases [10]. Africans display lower linkage disequilibrium and shorter haplotype blocks than other populations. These characteristics help with fine mapping of GWAS signals and identification of target genes, which are not only required to gain mechanistic insights, but may also inform therapeutic targets [89].

Only recently, through the Africa America Diabetes Mellitus (AADM) study [90], the Durban Diabetes Study (DDS) [91] and the Human Heredity and Health in Africa (H3Africa) Initiative [92], have GWAS of type 2 diabetes in sub-Saharan African populations been undertaken. A meta-analysis of 4347 sub-Saharan Africans showed that the variant most significantly associated with type 2 diabetes mapped to a locus near *TCF7L2*, replicating findings in other ethnic groups [93]. Fine mapping of *TCF7L2* revealed both an African-specific signal (rs17746147) and a signal shared with Europeans (rs7903146). The authors identified a novel African-specific association signal at *AGMO* (rs73284431) and 21 loci with shared causal variants in African and non-African populations [93]. Similarly, in a second GWAS using the AADM cohort, Adeyemo et al [94] showed transferability of 32 established type 2 diabetes loci, but also identified a novel locus for type 2 diabetes, namely *ZRANB3*, which has been shown to play a critical role in the production and maintenance of beta cells [94]. These findings highlight the importance of performing further adequately powered GWAS in SSA to identify novel risk loci to improve our understanding of the genetic architecture of type 2 diabetes in Africa. There are currently significant limitations to our understanding of the genetic underpinnings of certain traits, such as hepatic insulin clearance and body fat distribution, which appear to be specific and highly conserved in African populations. The increase of GWAS in SSA will enable the development of African-specific polygenic risk scores [95], which may refine type 2 diabetes risk prediction and provide greater understanding of the pathogenesis of type 2 diabetes in Africans.

Environmental factors also play a role in the aetiology of type 2 diabetes. Gene–environment interactions need to be considered, particularly in SSA where the genetic architecture, as well as environmental exposures, differs from those of European populations. The effects of the environment on type 2 diabetes risk may be mediated through epigenetic factors, such as DNA methylation, histone acetylation and microRNAs (miRNAs). In the first epigenome-wide association study (EWAS) in SSA, the team from the RODAM study identified four differentially methylated loci that were strongly and consistently associated with type 2 diabetes. Of these, three had been reported previously in other populations, but one differentially methylated locus was unique to the Ghanaian sample [96]. This again highlights the unique genetic architecture of Africans and the need for further EWAS to validate and extend these findings. More recently, a small study in South African women has shown that DNA methylation differs by ethnicity, obesity and adipose tissue depot [97]. Pheiffer et al profiled global and insulin receptor promotor DNA methylation in abdominal and gluteal SAT and revealed that global DNA methylation in gluteal SAT was associated with insulin resistance and systemic inflammation in Black South African women and not in White South African women [97]. These studies add to the body of work suggesting a specific role of gluteal SAT in type 2 diabetes risk in Black African women. As DNA methylation is reversible, identification of risk DNA methylation patterns may provide unique opportunities for intervention strategies.

## Conclusions

This review synthesises the evidence from SSA and shows that the pathogenesis of type 2 diabetes in Black Africans differs from the traditional model based on studies including participants of European ancestry and is mostly like that reported in diasporic Africans. We propose a model that highlights hyperinsulinaemia as the preeminent factor in the pathogenesis of type 2 diabetes in Black Africans (Fig. 4). However, there is a need for longitudinal and intervention studies to gain a complete understanding of the pathogenesis of type 2 diabetes in Black African men and women. Dietary interventions that reduce hyperinsulinaemia and obesity are recommended to gain insights into the mechanistic underpinnings of type 2 diabetes in this population.

## Supplementary Information

ESM 1 (PPTX 816 kb)

## Abbreviations

AADM: Africa America Diabetes Mellitus
ART: Anti-retroviral therapy
EWAS: Epigenome-wide association study
GWAS: Genome-wide association studies
IFG: Impaired fasting glucose
IGT: Impaired glucose tolerance
MVPA: Moderate-to-vigorous physical activity
NGT: Normal glucose tolerance
PLWH: People living with HIV
RODAM: Research on Obesity and Diabetes among African Migrants
SAT: Subcutaneous adipose tissue
SES: Socioeconomic status
SSA: Sub-Saharan Africa
VAT: Visceral adipose tissue
VLCD: Very low calorie diet

## References

[1] Saeedi P, Petersohn I, Salpea P et al (2019) Global and regional diabetes prevalence estimates for 2019 and projections for 2030 and 2045: Results from the International Diabetes Federation Diabetes Atlas, 9th edition. Diabetes Res Clin Pract 157:107843. 10.1016/j.diabres.2019.107843 10.1016/j.diabres.2019.107843 31518657

[2] Dillon DG, Gurdasani D, Riha J (2013). Association of HIV and ART with cardiometabolic traits in sub-Saharan Africa: a systematic review and meta-analysis. Int J Epidemiol.

[3] Ekoru K, Doumatey A, Bentley AR et al (2019) Type 2 diabetes complications and comorbidity in sub-Saharan Africans. EClinicalMedicine 16:30–41. 10.1016/j.eclinm.2019.09.001 10.1016/j.eclinm.2019.09.001 PMC6890980 31832618

[4] Goedecke JH, Olsson T (2020) Pathogenesis of type 2 diabetes risk in black Africans: a South African perspective. J Intern Med 288(3):284–294. 10.1111/joim.13083 10.1111/joim.13083 32303113

[5] Utumatwishima JN, Chung ST, Bentley AR, Udahogora M, Sumner AE (2018). Reversing the tide - diagnosis and prevention of T2DM in populations of African descent. Nat Rev Endocrinol.

[6] Gaillard TR, Osei K (2016). Racial Disparities in the Pathogenesis of Type 2 Diabetes and its Subtypes in the African Diaspora: A New Paradigm. J Racial Ethn Health Disparities.

[7] Kodama K, Tojjar D, Yamada S, Toda K, Patel CJ, Butte AJ (2013). Ethnic differences in the relationship between insulin sensitivity and insulin response: a systematic review and meta-analysis. Diabetes Care.

[8] Goff LM, Ladwa M, Hakim O, Bello O (2019). Ethnic distinctions in the pathophysiology of type 2 diabetes: a focus on black African-Caribbean populations. Proc Nutr Soc.

[9] Ladwa M, Hakim O, Amiel SA, Goff LM (2019) A systematic review of beta cell function in adults of black African ethnicity. J Diabetes Res 2019:7891359. 10.1155/2019/7891359 10.1155/2019/7891359 PMC6855028 31781667

[10] Pereira L, Mutesa L, Tindana P, Ramsay M (2021). African genetic diversity and adaptation inform a precision medicine agenda. Nat Rev Genet.

[11] Esser N, Utzschneider KM, Kahn SE (2020). Early beta cell dysfunction vs insulin hypersecretion as the primary event in the pathogenesis of dysglycaemia. Diabetologia.

[12] Petersen MC, Shulman GI (2018). Mechanisms of Insulin Action and Insulin Resistance. Physiol Rev.

[13] Trico D, Natali A, Arslanian S, Mari A, Ferrannini E (2018). Identification, pathophysiology, and clinical implications of primary insulin hypersecretion in nondiabetic adults and adolescents. JCI Insight.

[14] Bergman RN, Piccinini F, Kabir M, Kolka CM, Ader M (2019). Hypothesis: Role of Reduced Hepatic Insulin Clearance in the Pathogenesis of Type 2 Diabetes. Diabetes.

[15] Goedecke JH, Dave JA, Faulenbach MV (2009). Insulin response in relation to insulin sensitivity: an appropriate beta-cell response in black South African women. Diabetes Care.

[16] Osei K, Schuster DP, Owusu SK, Amoah AG (1997). Race and ethnicity determine serum insulin and C-peptide concentrations and hepatic insulin extraction and insulin clearance: comparative studies of three populations of West African ancestry and white Americans. Metabolism.

[17] Gower BA, Fowler LA (2020) Obesity in African-Americans: the role of physiology. J Intern Med. 10.1111/joim.13090 10.1111/joim.13090 32350924

[18] Ladwa M, Bello O, Hakim O (2020). Insulin clearance as the major player in the hyperinsulinaemia of black African men without diabetes. Diabetes Obes Metab.

[19] Chung ST, Galvan-De La Cruz M, Aldana PC (2019). Postprandial insulin response and clearance among black and white women: the federal women's study. J Clin Endocrinol Metab.

[20] Fortuin-de Smidt MC, Mendham AE, Hauksson J (2021). beta-cell function in black South African women: exploratory associations with insulin clearance, visceral and ectopic fat. Endocr Connect.

[21] Amoah AG, Owusu SK, Schuster DP, Osei K (2002). Pathogenic mechanism of type 2 diabetes in Ghanaians--the importance of beta cell secretion, insulin sensitivity and glucose effectiveness. S Afr Med J.

[22] Goedecke JH, George C, Veras K (2016). Sex differences in insulin sensitivity and insulin response with increasing age in black South African men and women. Diabetes Res Clin Pract.

[23] PrayGod G, Filteau S, Range N (2021). beta-cell dysfunction and insulin resistance in relation to pre-diabetes and diabetes among adults in north-western Tanzania: a cross-sectional study. Tropical Med Int Health.

[24] Meeks KAC, Stronks K, Adeyemo A (2017). Peripheral insulin resistance rather than beta cell dysfunction accounts for geographical differences in impaired fasting blood glucose among sub-Saharan African individuals: findings from the RODAM study. Diabetologia.

[25] Kengne AP, Erasmus RT, Levitt NS, Matsha TE (2017). Alternative indices of glucose homeostasis as biochemical diagnostic tests for abnormal glucose tolerance in an African setting. Prim Care Diabetes.

[26] Alford FP, Henriksen JE, Rantzau C, Beck-Nielsen H (2018). Glucose effectiveness is a critical pathogenic factor leading to glucose intolerance and type 2 diabetes: An ignored hypothesis. Diabetes Metab Res Rev.

[27] Osei K, Schuster DP (1996). Effects of race and ethnicity on insulin sensitivity, blood pressure, and heart rate in three ethnic populations: comparative studies in African-Americans, African immigrants (Ghanaians), and white Americans using ambulatory blood pressure monitoring. Am J Hypertens.

[28] Goedecke JH, Levitt NS, Lambert EV (2009). Differential effects of abdominal adipose tissue distribution on insulin sensitivity in black and white South African women. Obesity (Silver Spring).

[29] Keswell D, Tootla M, Goedecke JH (2016). Associations between body fat distribution, insulin resistance and dyslipidaemia in black and white South African women. Cardiovasc J Afr.

[30] Goedecke JH, Evans J, Keswell D (2011). Reduced gluteal expression of adipogenic and lipogenic genes in Black South African women is associated with obesity-related insulin resistance. J Clin Endocrinol Metab.

[31] Kotze-Horstmann LM, Keswell D, Adams K, Dlamini T, Goedecke JH (2017). Hypoxia and extra-cellular matrix gene expression in adipose tissue associates with reduced insulin sensitivity in black South African women. Endocrine.

[32] Evans J, Goedecke JH, Soderstrom I (2011). Depot- and ethnic-specific differences in the relationship between adipose tissue inflammation and insulin sensitivity. Clin Endocrinol.

[33] Mendham AE, Larsen S, George C (2020). Exercise training results in depot-specific adaptations to adipose tissue mitochondrial function. Sci Rep.

[34] Nono Nankam PA, Bluher M, Kehr S (2020). Distinct abdominal and gluteal adipose tissue transcriptome signatures are altered by exercise training in African women with obesity. Sci Rep.

[35] Reed RM, Nevitt SJ, Kemp GJ, Cuthbertson DJ, Whyte MB, Goff LM (2022) Ectopic fat deposition in populations of black African ancestry: A systematic review and meta-analysis. Acta Diabetol 59:171–187. 10.1007/s00592-021-01797-5 10.1007/s00592-021-01797-5 PMC8841318 34518896

[36] Goedecke JH, Keswell D, Weinreich C (2015). Ethnic differences in hepatic and systemic insulin sensitivity and their associated determinants in obese black and white South African women. Diabetologia.

[37] Goedecke JH, Chorell E, van Jaarsveld PJ, Riserus U, Olsson T (2021) Fatty acid metabolism and associations with insulin sensitivity differs between black and white South African women. J Clin Endocrinol Metab 106(1):e140–e151. 10.1210/clinem/dgaa696 10.1210/clinem/dgaa696 32995848

[38] Chung ST, Courville AB, Onuzuruike AU (2018). Gluconeogenesis and risk for fasting hyperglycemia in Black and White women. JCI Insight.

[39] Knight MG, Goedecke JH, Ricks M (2011). The TG/HDL-C ratio does not predict insulin resistance in overweight women of African descent: a study of South African, African American and West African women. Ethn Dis.

[40] Mtintsilana A, Micklesfield LK, Chorell E, Olsson T, Goedecke JH (2019). Fat redistribution and accumulation of visceral adipose tissue predicts type 2 diabetes risk in middle-aged black South African women: a 13-year longitudinal study. Nutr Diabetes.

[41] van Dijk AM, Dingerink S, Chilunga FP et al (2021) Metabolic-associated fatty liver disease as assessed by the fatty liver index among migrant and non-migrant Ghanaian populations. J Clin Transl Hepatol 9(4):494–502. 10.14218/JCTH.2021.00066 10.14218/JCTH.2021.00066 PMC8369017 34447678

[42] Ng M, Fleming T, Robinson M (2014). Global, regional, and national prevalence of overweight and obesity in children and adults during 1980–2013: a systematic analysis for the Global Burden of Disease Study 2013. Lancet.

[43] Kufe C, Micklesfield LK, Masemola M et al (2022) Increased risk for type 2 diabetes in relation to adiposity in middle-aged black South African men compared to women. Eur J Endocrinol. 10.1530/eje-21-0527 10.1530/EJE-21-0527 PMC9010812 35225824

[44] Micklesfield LK, Kagura J, Munthali R et al (2018) Demographic, socio-economic and behavioural correlates of BMI in middle-aged black men and women from urban Johannesburg, South Africa. Glob Health Action 11(Suppl 2):1448250. 10.1080/16549716.2018.1448250 10.1080/16549716.2018.1448250 PMC6084500 30079826

[45] Boua PR, Soo CC, Debpuur C (2021). Prevalence and socio-demographic correlates of tobacco and alcohol use in four sub-Saharan African countries: a cross-sectional study of middle-aged adults. BMC Public Health.

[46] Kufe C, Goedecke JH, Masemola M et al (2022) Physical behaviours and their association with type 2 diabetes mellitus risk markers in urban South African middle-aged adults: An isotemporal substitution approach BMJ Open Diabetes Res Care 10(4):e002815. 10.1136/bmjdrc-2022-002815 10.1136/bmjdrc-2022-002815 PMC9280902 35831028

[47] Ratshikombo T, Goedecke JH, Soboyisi M et al (2021) Sex differences in the associations of nutrient patterns with total and regional adiposity: a study of middle-aged black South African men and women. Nutrients 13(12):4558. 10.3390/nu13124558 10.3390/nu13124558 PMC8706560 34960108

[48] Lim EL, Hollingsworth KG, Aribisala BS, Chen MJ, Mathers JC, Taylor R (2011). Reversal of type 2 diabetes: normalisation of beta cell function in association with decreased pancreas and liver triacylglycerol. Diabetologia.

[49] Fortuin-de Smidt MC, Mendham AE, Hauksson J et al (2020) Effect of exercise training on insulin sensitivity, hyperinsulinemia and ectopic fat in black South African women: A randomized controlled trial. Eur J Endocrinol 183(1):51–61. 10.1530/eje-19-0957 10.1530/EJE-19-0957 32503004

[50] Gower BA, Chandler-Laney PC, Ovalle F (2013). Favourable metabolic effects of a eucaloric lower-carbohydrate diet in women with PCOS. Clin Endocrinol.

[51] Arad AD, DiMenna FJ, Thomas N (2015). High-intensity interval training without weight loss improves exercise but not basal or insulin-induced metabolism in overweight/obese African American women. J Appl Physiol (1985).

[52] Goedecke JH, Mendham AE, Clamp L et al (2018) An exercise intervention to unravel the mechanisms underlying insulin resistance in a cohort of black South African women: protocol for a randomized controlled trial and baseline characteristics of participants. JMIR Res Protoc 7(4):e75. 10.2196/resprot.9098 10.2196/resprot.9098 PMC5932332 29669711

[53] Samuel-Hodge CD, Johnson CM, Braxton DF, Lackey M (2014). Effectiveness of diabetes prevention program translations among African Americans. Obes Rev.

[54] Malin SK, Solomon TP, Blaszczak A, Finnegan S, Filion J, Kirwan JP (2013). Pancreatic beta-cell function increases in a linear dose-response manner following exercise training in adults with prediabetes. Am J Physiol Endocrinol Metab.

[55] Slentz CA, Tanner CJ, Bateman LA (2009). Effects of exercise training intensity on pancreatic beta-cell function. Diabetes Care.

[56] Nono Nankam PA, Mendham AE, De Smidt MF (2020). Changes in systemic and subcutaneous adipose tissue inflammation and oxidative stress in response to exercise training in obese black African women. J Physiol.

[57] Mendham AE, Goedecke JH, Zeng Y (2021). Exercise training improves mitochondrial respiration and is associated with an altered intramuscular phospholipid signature in women with obesity. Diabetologia.

[58] Gower BA, Goss AM (2015). A lower-carbohydrate, higher-fat diet reduces abdominal and intermuscular fat and increases insulin sensitivity in adults at risk of type 2 diabetes. J Nutr.

[59] Chen M, Moran LJ, Harrison CL (2022). Ethnic differences in response to lifestyle intervention for the prevention of type 2 diabetes in adults: A systematic review and meta-analysis. Obes Rev.

[60] Bynoe K, Unwin N, Taylor C (2020). Inducing remission of Type 2 diabetes in the Caribbean: findings from a mixed methods feasibility study of a low-calorie liquid diet-based intervention in Barbados. Diabet Med.

[61] Atun R, Davies JI, Gale EAM (2017). Diabetes in sub-Saharan Africa: from clinical care to health policy. Lancet Diabetes Endocrinol.

[62] Addo J, Agyemang C, de-Graft Aikins A (2017). Association between socioeconomic position and the prevalence of type 2 diabetes in Ghanaians in different geographic locations: the RODAM study. J Epidemiol Community Health.

[63] Bavuma CM, Musafiri S, Rutayisire PC, Ng'ang'a LM, McQuillan R, Wild SH (2020). Socio-demographic and clinical characteristics of diabetes mellitus in rural Rwanda: time to contextualize the interventions? A cross-sectional study. BMC Endocr Disord.

[64] Steyn NP, McHiza ZJ (2014) Obesity and the nutrition transition in sub-Saharan Africa. Ann N Y Acad Sci 1311:88–101. 10.1111/nyas.12433 10.1111/nyas.12433 24725148

[65] McHiza ZJ, Steyn NP, Hill J (2015). A review of dietary surveys in the adult South African population from 2000 to 2015. Nutrients.

[66] Galbete C, Nicolaou M, Meeks K (2018). Dietary patterns and type 2 diabetes among Ghanaian migrants in Europe and their compatriots in Ghana: the RODAM study. Nutr Diabetes.

[67] Danquah I, Addo J, Boateng D (2019). Early-life factors are associated with waist circumference and type 2 diabetes among Ghanaian adults: The RODAM Study. Sci Rep.

[68] Norris SA, Osmond C, Gigante D (2012). Size at birth, weight gain in infancy and childhood, and adult diabetes risk in five low- or middle-income country birth cohorts. Diabetes Care.

[69] Levitt NS, Lambert EV, Woods D, Hales CN, Andrew R, Seckl JR (2000) Impaired glucose tolerance and elevated blood pressure in low birth weight, nonobese, young South African adults: early programming of cortisol axis. J Clin Endocrinol Metab 85(12):4611–4618. 10.1210/jcem.85.12.7039 10.1210/jcem.85.12.7039 11134116

[70] Crowther NJ, Cameron N, Trusler J, Toman M, Norris SA, Gray IP (2008). Influence of catch-up growth on glucose tolerance and beta-cell function in 7-year-old children: results from the birth to twenty study. Pediatrics.

[71] Filteau S, PrayGod G, Rehman AM (2021). Prior undernutrition and insulin production several years later in Tanzanian adults. Am J Clin Nutr.

[72] Bavuma C, Sahabandu D, Musafiri S, Danquah I, McQuillan R, Wild S (2019). Atypical forms of diabetes mellitus in Africans and other non-European ethnic populations in low- and middle-income countries: a systematic literature review. J Glob Health.

[73] Kibirige D, Sekitoleko I, Lumu W (2022). Understanding the pathogenesis of lean non-autoimmune diabetes in an African population with newly diagnosed diabetes. Diabetologia.

[74] Kweka BV, Fredrick C, Kitilya B (2022). Association of sickle cell trait with beta-cell dysfunction and physical activity in adults living with and without HIV in Tanzania. APMIS.

[75] Wamai RG, Shirley HC, Greiner C, Van Wolputte S, Bollig M (2022). The future of health in sub-Saharan Africa: is there a path to longer and healthier lives for all?. African Futures.

[76] LeCroix RH, Chan WY, Henrich C, Palin F, Shanley J, Armistead L (2019) Maternal HIV and adolescent functioning in South Africa: the role of the mother-child relationship. J Early Adolesc 40(1):83–103. 10.1177/0272431618824726

[77] Dwyer-Lindgren L, Cork MA, Sligar A (2019). Mapping HIV prevalence in sub-Saharan Africa between 2000 and 2017. Nature.

[78] Zhang S, Carper MJ, Lei X, Cade WT, Yarasheski KE, Ramanadham S (2009). Protease inhibitors used in the treatment of HIV+ induce beta-cell apoptosis via the mitochondrial pathway and compromise insulin secretion. Am J Physiol Endocrinol Metab.

[79] Prioreschi A, Munthali RJ, Soepnel L (2017). Incidence and prevalence of type 2 diabetes mellitus with HIV infection in Africa: a systematic review and meta-analysis. BMJ Open.

[80] Lagathu C, Béréziat V, Gorwood J (2019). Metabolic complications affecting adipose tissue, lipid and glucose metabolism associated with HIV antiretroviral treatment. Expert Opin Drug Saf.

[81] Venter WDF, Sokhela S, Simmons B (2020). Dolutegravir with emtricitabine and tenofovir alafenamide or tenofovir disoproxil fumarate versus efavirenz, emtricitabine, and tenofovir disoproxil fumarate for initial treatment of HIV-1 infection (ADVANCE): week 96 results from a randomised, phase 3, non-inferiority trial. Lancet HIV.

[82] Layden JE, Phillips R, Opare-Sem O et al (2014) Hepatitis C in sub-Saharan Africa: urgent need for attention. Open Forum Infect Dis 1(2):ofu065. 10.1093/ofid/ofu065 10.1093/ofid/ofu065 PMC4281810 25734135

[83] Fabiani S, Fallahi P, Ferrari SM, Miccoli M, Antonelli A (2018). Hepatitis C virus infection and development of type 2 diabetes mellitus: systematic review and meta-analysis of the literature. Rev Endocr Metab Disord.

[84] Sobngwi E, Choukem SP, Agbalika F et al (2008) Ketosis-prone type 2 diabetes mellitus and human herpesvirus 8 infection in sub-Saharan Africans. JAMA 299(23):2770–2776. 10.1001/jama.299.23.2770 10.1001/jama.299.23.2770 18560004

[85] Nguewa JL, Lontchi-Yimagou E, Agbelika F (2017). Relationship between HHV8 infection markers and insulin sensitivity in ketosis-prone diabetes. Diabetes Metab.

[86] Lontchi-Yimagou E, Legoff J, Nguewa JL (2018). Human herpesvirus 8 infection DNA positivity is associated with low insulin secretion: A case-control study in a sub-Saharan African population with diabetes. J Diabetes.

[87] Tracey EF, McDermott RA, McDonald MI (2016). Do worms protect against the metabolic syndrome? A systematic review and meta-analysis. Diabetes Res Clin Pract.

[88] PrayGod G, Filteau S, Range N (2022). The association of Schistosoma and geohelminth infections with beta-cell function and insulin resistance among HIV-infected and HIV-uninfected adults: A cross-sectional study in Tanzania. PLoS One.

[89] Fatumo S, Chikowore T, Choudhury A, Ayub M, Martin AR, Kuchenbaecker K (2022). A roadmap to increase diversity in genomic studies. Nat Med.

[90] Rotimi CN, Chen G, Adeyemo AA (2004). A genome-wide search for type 2 diabetes susceptibility genes in West Africans: the Africa America Diabetes Mellitus (AADM) Study. Diabetes.

[91] Hird TR, Young EH, Pirie FJ (2016). Study profile: the Durban Diabetes Study (DDS): a platform for chronic disease research. Glob Health Epidemiol Genom.

[92] Ekoru K, Young EH, Adebamowo C (2016). H3Africa multi-centre study of the prevalence and environmental and genetic determinants of type 2 diabetes in sub-Saharan Africa: study protocol. Glob Health Epidemiol Genom.

[93] Chen J, Sun M, Adeyemo A (2019). Genome-wide association study of type 2 diabetes in Africa. Diabetologia.

[94] Adeyemo AA, Zaghloul NA, Chen G (2019). ZRANB3 is an African-specific type 2 diabetes locus associated with beta-cell mass and insulin response. Nat Commun.

[95] Chikowore T, Ekoru K, Vujkovi M (2022). Polygenic prediction of type 2 diabetes in Africa. Diabetes Care.

[96] Meeks KAC, Henneman P, Venema A (2019). Epigenome-wide association study in whole blood on type 2 diabetes among sub-Saharan African individuals: findings from the RODAM study. Int J Epidemiol.

[97] Pheiffer C, Willmer T, Dias S, Abrahams Y, Louw J, Goedecke JH (2020). Ethnic and adipose depot specific associations between DNA methylation and metabolic risk. Front Genet.

[98] Clamp LD, Mendham AE, Kroff J, Goedecke JH (2020). Higher baseline fat oxidation promotes gynoid fat mobilization in response to a 12-week exercise intervention in sedentary, obese black South African women. Appl Physiol Nutr Metab.

[99] Nono Nankam PA, Mendham AE, van Jaarsveld PJ et al (2020) Exercise training alters red blood cell fatty acid desaturase indices and adipose tissue fatty acid profile in African women with obesity. Obesity (Silver Spring) 28(8):1456–1466. 10.1002/oby.22862 10.1002/oby.22862 32627952

