# Supplementary material for: Pathophysiology of type 2 diabetes in sub-Saharan Africans
Source: Diabetologia. 2022 Sep 27;65(12):1967–80. doi: 10.1007/s00125-022-05795-2 (PMC9630207; doi:10.1007/s00125-022-05795-2)
Supplement: Supplementary file 1 — (PPTX 816 kb) [file 125_2022_5795_MOESM1_ESM.pptx]

## Slide 1
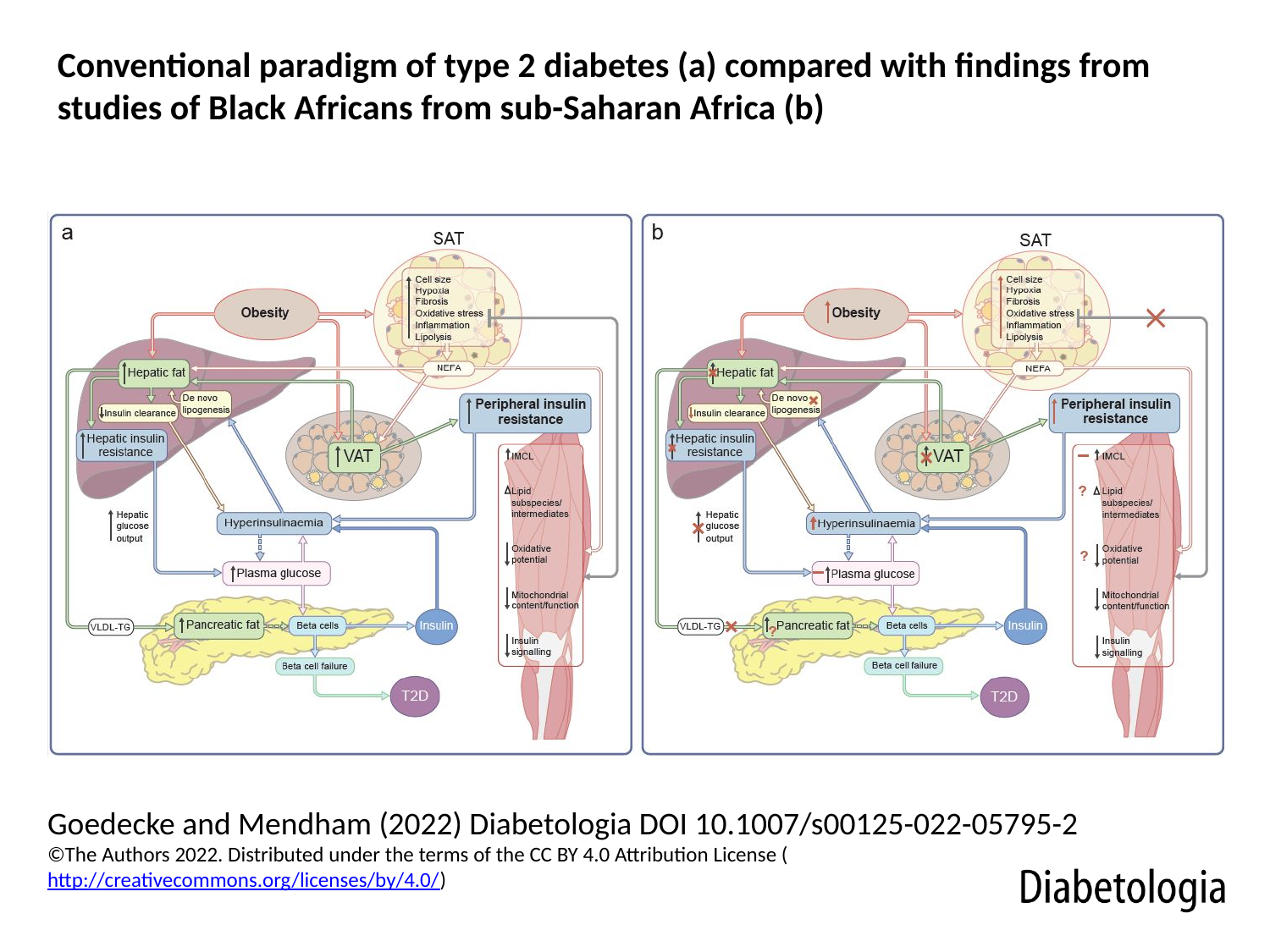

Conventional paradigm of type 2 diabetes (a) compared with findings from studies of Black Africans from sub-Saharan Africa (b)
Goedecke and Mendham (2022) Diabetologia DOI 10.1007/s00125-022-05795-2
©The Authors 2022. Distributed under the terms of the CC BY 4.0 Attribution License (http://creativecommons.org/licenses/by/4.0/)

## Slide 2
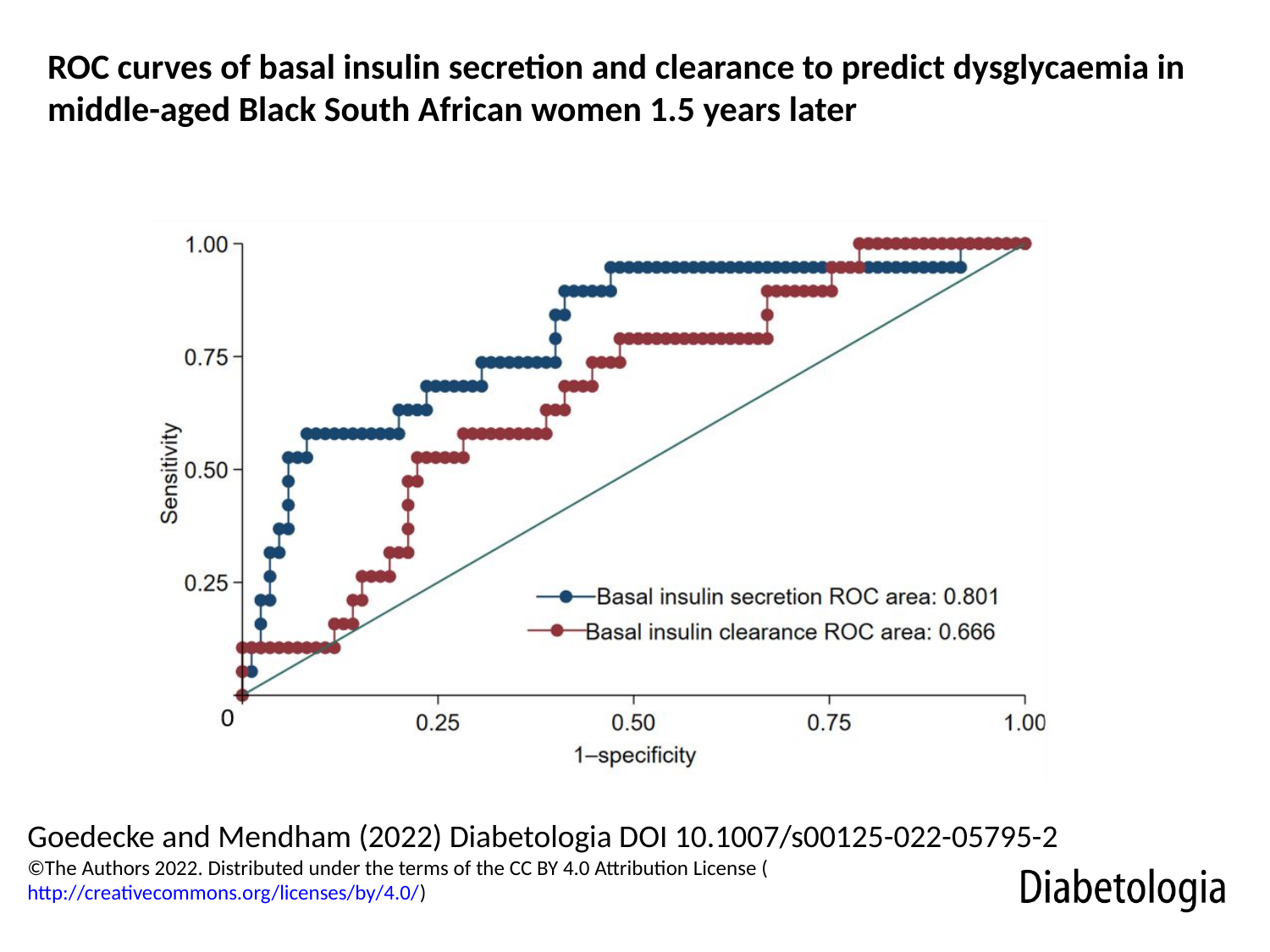

ROC curves of basal insulin secretion and clearance to predict dysglycaemia in middle-aged Black South African women 1.5 years later
Goedecke and Mendham (2022) Diabetologia DOI 10.1007/s00125-022-05795-2
©The Authors 2022. Distributed under the terms of the CC BY 4.0 Attribution License (http://creativecommons.org/licenses/by/4.0/)

## Slide 3
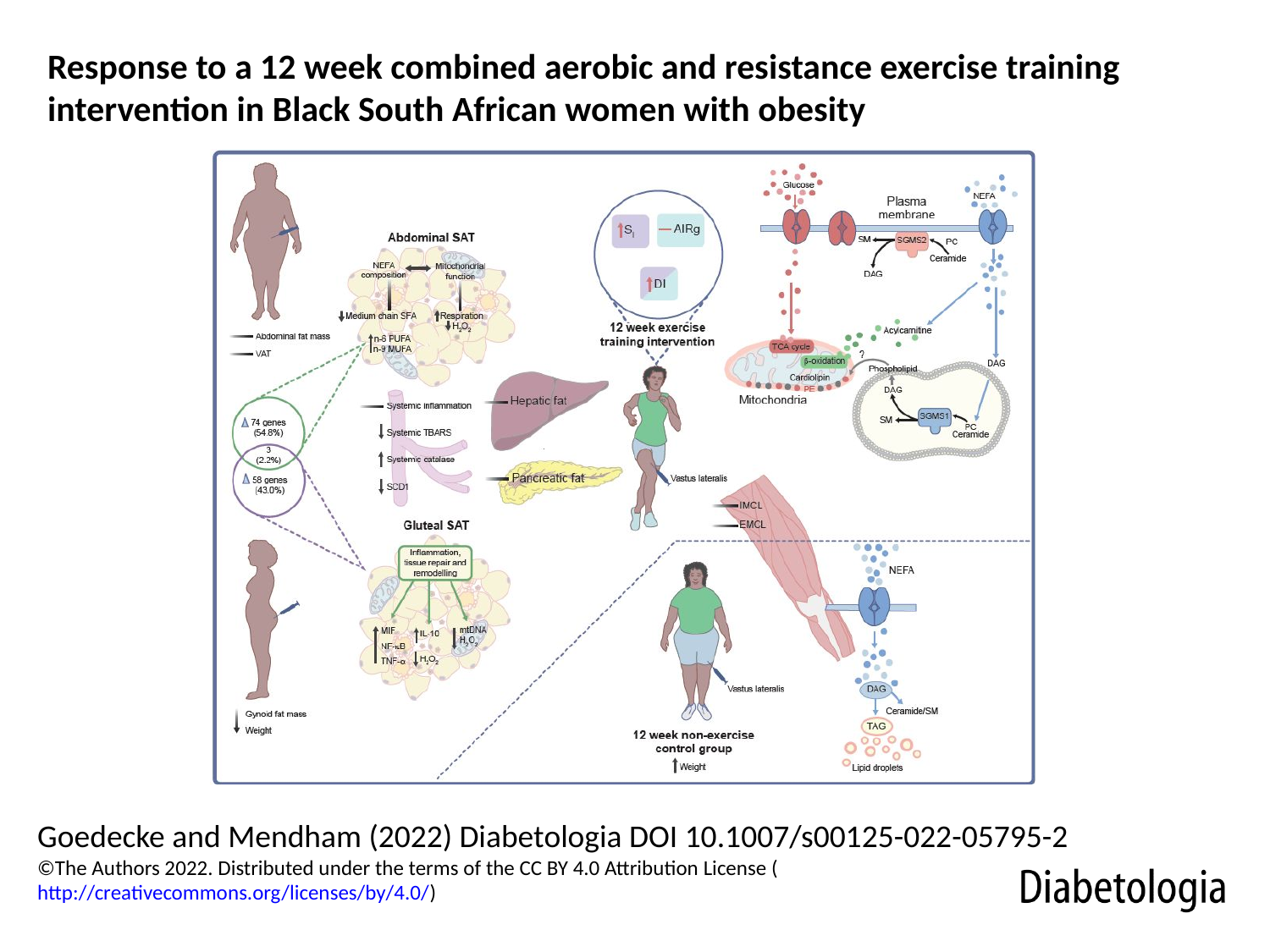

Response to a 12 week combined aerobic and resistance exercise training intervention in Black South African women with obesity
Goedecke and Mendham (2022) Diabetologia DOI 10.1007/s00125-022-05795-2
©The Authors 2022. Distributed under the terms of the CC BY 4.0 Attribution License (http://creativecommons.org/licenses/by/4.0/)

## Slide 4
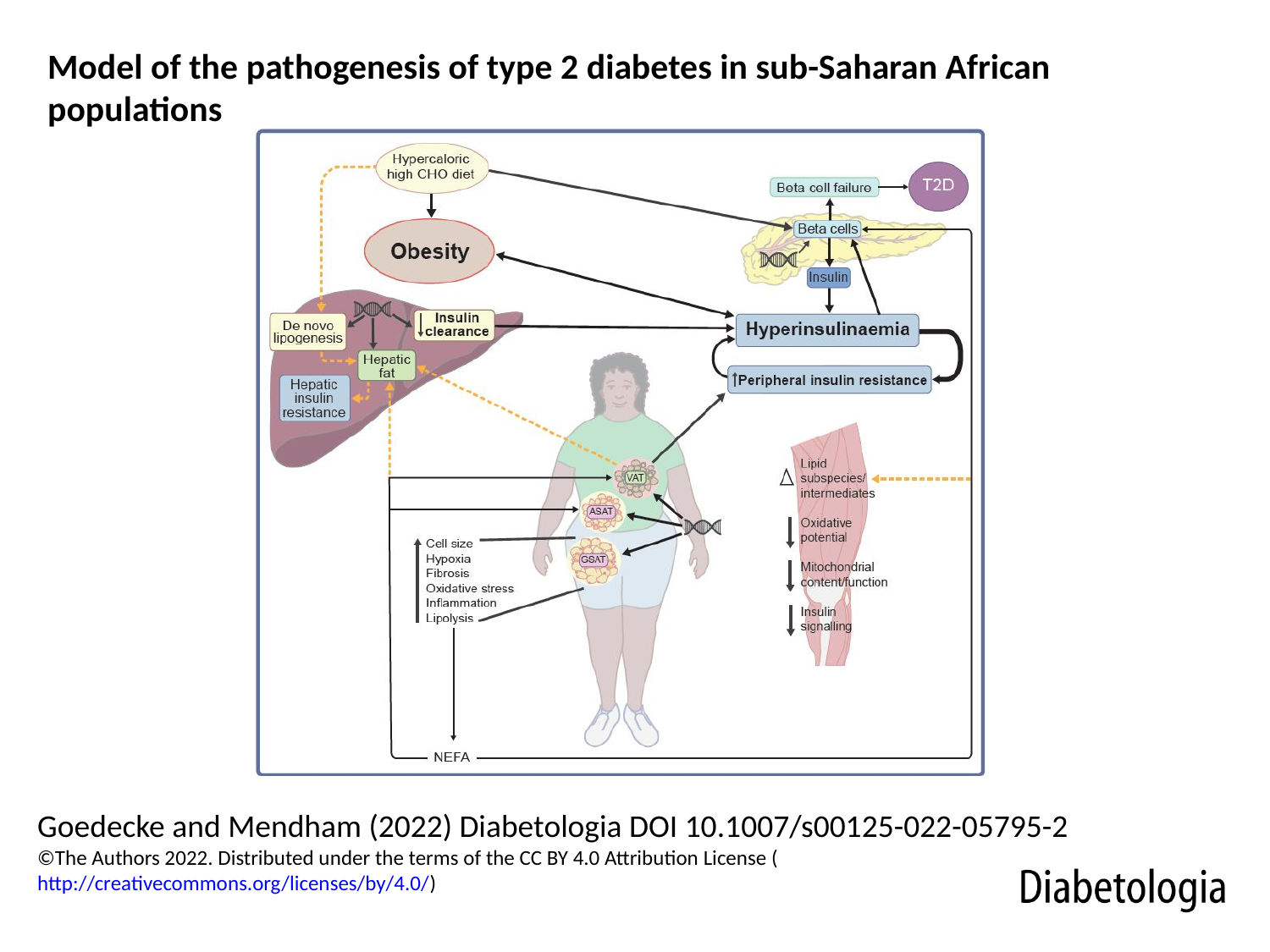

Model of the pathogenesis of type 2 diabetes in sub-Saharan African populations
Goedecke and Mendham (2022) Diabetologia DOI 10.1007/s00125-022-05795-2
©The Authors 2022. Distributed under the terms of the CC BY 4.0 Attribution License (http://creativecommons.org/licenses/by/4.0/)
